# Spontaneous Pneumomediastinum in a Middle-Aged Female Patient With Schizophrenia: A Case Report

**DOI:** 10.7759/cureus.51887

**Published:** 2024-01-08

**Authors:** Takahiko Nagamine

**Affiliations:** 1 Psychosomatic Dentistry, Tokyo Medical and Dental University, Tokyo, JPN; 2 Psychiatric Internal Medicine, Sunlight Brain Research Center, Yamaguchi, JPN

**Keywords:** delusion, vacuum phenomenon, mediastinal pressure, schizophrenia, spontaneous pneumomediastinum

## Abstract

A middle-aged female patient with schizophrenia and osteoporosis presented to the emergency department with complaints of sore throat, neck pain, and dysphagia, which was identified as spontaneous pneumomediastinum (SPM) on chest CT. SPM has been reported in anorexia nervosa, but this is the first report of SPM in schizophrenia. In anorexia nervosa, an increase in intrathoracic pressure because of vomiting can cause positive pressure SPM, but this patient was considered to have negative pressure SPM because of decreased mediastinal pressure. In schizophrenia patients with unexplained chest pain, neck pain, and dysphagia, SPM should be considered a differential disease, and a chest CT scan is useful for diagnosis.

## Introduction

Spontaneous pneumomediastinum (SPM) is a rare condition wherein air is present in the mediastinum without a significant cause, primarily affecting young males [1]. The mechanism of SPM is thought to be that the alveoli rupture because of increased intrathoracic pressure, and air accumulates in the mediastinum along the bronchi. The symptoms are often ambiguous, such as chest pain, neck pain, and difficulty swallowing, making diagnosis difficult [1]. We examined a middle-aged female patient with schizophrenia whose neck pain and dysphagia were caused by SPM. SPM associated with psychiatric disorders has been reported in anorexia nervosa [2], but this is the first case reported in schizophrenia. The absence of increased intrathoracic pressure in this patient suggests a different mechanism for the pathogenesis of SPM than what has been previously reported.

## Case presentation

**Figure 1:**
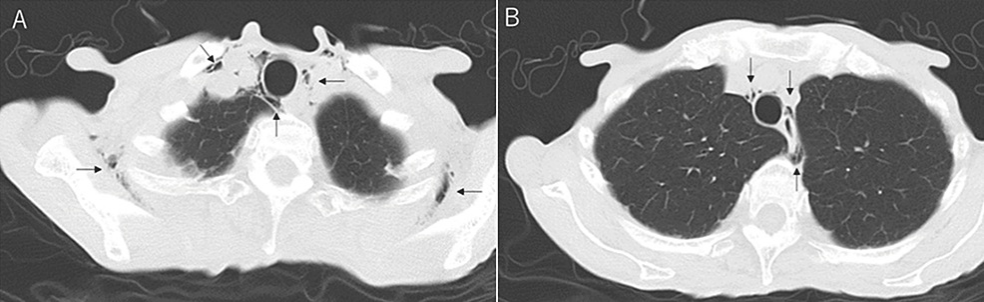
Chest computed tomography The image near the lung apex shows free air in the mediastinum and subcutaneous emphysema in the soft tissues of the back (A). Free air can be seen from the spine to the mediastinum surrounding the bronchi (B).

A 60-year-old female patient, with schizophrenia and osteoporosis who was being treated with olanzapine 20 mg/day and eldecalcitol 0.75 μg/day, visited a psychiatrist five days earlier complaining of neck and chest pain and difficulty swallowing and claimed that her food was poisoned. She was suspected of a relapse of delusion, but because she was unable to eat for several days, she was transferred to the emergency department. She was thin with a BMI of 17.1 kg/m^2^ (height 153 cm, weight 40 kg). She was a non-smoker and had no history of drug use. She had no history of asthma, COPD, or other lung diseases causing secondary pneumomediastinum. On arrival, her consciousness was clear, and her vital signs were stable. Although her chest pain had resolved, she still had a sore throat and difficulty swallowing. The electrocardiogram was within normal limits, and the chest X-ray showed no obvious abnormalities. Upper gastrointestinal endoscopy revealed no obvious abnormalities. Blood tests also showed a white blood cell count within normal limits and a negative C-reactive protein. However, a chest CT scan showed free air in the normal-sized mediastinum and subcutaneous emphysema in the soft tissue of the dorsal upper chest, leading to the diagnosis of SPM (Figure 1).

The patient was admitted to our hospital and managed with intravenous fluids. On day three of hospitalization, her dysphagia and neck pain disappeared, and she was able to eat. Once the food began to pass down her throat, the delusion that the food was poisoned disappeared. On day five of hospitalization, she was discharged after a chest x-ray showed no abnormalities and no recurrence of SPM. At a follow-up one month after discharge, she had no delusions and was eating well.

## Discussion

This is the first case of SPM in a patient with schizophrenia who presented with neck pain, chest pain, and dysphagia. Because of dysphagia, the patient developed delusions of poison in her food, but these delusions decreased with the improvement of SPM. When the patient came to our hospital, it had been five days since the chest pain, and then SPM had already begun to be absorbed, making it difficult to detect mediastinal or subcutaneous emphysema on chest X-ray. If her SPM had not been diagnosed without performing a chest CT scan, the patient's delusions could have been even more intense. In patients with schizophrenia, it is difficult to assess physical symptoms present along with psychotic symptoms. Chest CT scans can be useful in detecting organic causes of dysphagia and chest symptoms, as seen in a report where chest CT detected a tracheal foreign body in a patient with schizophrenia [3].

In clinical psychiatry, SPM has been reported in patients with anorexia nervosa, who often experience repeated vomiting, so increased intrathoracic pressure is thought to be the mechanism of onset [2]. When air pressure in the alveoli rapidly increases during vomiting, free air leaks from ruptured alveoli and accumulates along the bronchovascular tissue sheath, and then free air moves toward the mediastinum. In this patient, chest pain five days ago and the presence of subcutaneous emphysema may be consistent with air leaks from ruptured alveoli. However, the patient was a thin, middle-aged female with little subcutaneous adipose tissue, subcutaneous emphysema limited to the back, mild chest pain, and no event that could have caused the increased intrathoracic pressure. Moreover, there was no mediastinal dilatation on chest CT. Thus, the air in this patient's mediastinum may have been inflated with some gas to fill the low-pressure gap.

SPM can be classified into two categories, positive pressure SPM and negative pressure SPM [4]. Positive pressure SPM is conventionally referred to as an air leak because of increased intrathoracic pressure, while negative pressure SPM is an influx of gas to the mediastinum because of decreased mediastinum pressure. An example of a source of gas is the vacuum phenomenon, which is the asymptomatic accumulation of gas in a degenerated intervertebral disc [5]. This patient may have negative pressure SPM because of the presence of space in the tissues that would be affected by emaciation, the possibility of spinal vacuum phenomenon because of osteoporosis, and the absence of mediastinal enlargement findings on chest CT.

## Conclusions

SPM is a rare disorder characterized by the presence of free air in the mediastinum despite the absence of a causative agent. This patient is the first report of SPM in schizophrenia and differs from previous reports of SPM in the following two respects: (1) the age of onset is higher than the usual age of onset, and (2) the mechanism of onset is the change in mediastinum pressure and gas influx. Although SPM is considered a disease of male adolescents, thin middle-aged female patients are also at risk because of decreased mediastinal pressure. In schizophrenia patients with unexplained chest pain, neck pain, and dysphagia, SPM should be considered as a differential disease, and a chest CT scan is useful for diagnosis.
